# Operative Procedures in Glandular Tumors of the Neck

**Published:** 1895-01

**Authors:** J. E. Thompson

**Affiliations:** M. D., B. S., London Univ.; F. R. C. S., England


For Texas Medical Journal.

### OPERATIVE PROCEDURES IN GLANDULAR TUMORS OF THE NECK.

BY J. E. THOMPSON, M. D., B. S., LOND. UNIV.; F. R. C. S., ENG.

[Read before the Galveston County Medical Society.]

OPERATIVE procedures in glandular tumors of the neck are pregnant with such dangers and full of such interesting problems, that they form at once the most dangerous and the most enticing work an enthusiastic surgeon comes in contact with. Oftentimes it is exceedingly difficult to see a successful termination to an operation commenced under the most favorable auspices, and again an unpromising tumor shells out like an orange from its skin. Familiarity with the pathology of these growths is essential; often more important than a knowledge of anatomy, for it would be manifestly dangerous to attack an aneurism of the carotid under the belief that it was a pulsating adenoma of the thyroid, while many cases of lympho sarcoma are utterly beyond the reach of human aid, where a simple lymphadenoma or tubercular enlargement of the same size might be successfully dealt with.

But pathology, however necessary it may be, is not everything. Many cases present themselves where we find it impossible to diagnose between lymphadenoma and tuberculous glands (Cf. case 2). In case 2 examination of the blood revealed a decided increase in the number of white corpuscles, and a coincident enlargement of the glands in both axillæ led me to suppose that I had a general lymphatic affection, but operation showed an enormous mass of tubercular glands, extending from the mastoid process to the dome of the pleura.

The cases which follow will bring out the various points I wish to emphasize.

CASE I.—An Italian girl, aged 7, was brought under my notice in June last, having an enormous mass of glands in the left side of the neck, along the course of the carotid artery, extending from the hyoid bone above to the supra-clavicular space below, completely filling the posterior triangle. The tumor showed a depression corresponding to the sterno-mastoid muscle. The glands were nodular, freely movable under the skin, and fairly movable in a transverse direction from the middle line. The child was anæmic, but there was no appreciable increase in the number of white blood corpuscles, nor was there any enlargement of liver or spleen. Temperature was a little elevated in the evening. I made a diagnosis of lymphadenoma (Hodgkins' disease), and advised removal.

The operation was commenced by an incision along the anterior border of sterno mastoid, extending from the sternum to the hyoid bone; another being made from the middle of this, at right angles, across the posterior triangle of the neck, cutting through the sterno mastoid. I then exposed the common carotid artery and internal jugular vein, and proceeded to isolate them from the growth along its whole length. This was tedious and difficult, as the glands were rather adherent to the sheath of the jugular. The vagus nerve was exposed along the whole field of the operation, and at the lower part of the neck, as I was enucleating some glands which extended rather deeply into the thorax, a spurt of chylous fluid occurred. I had torn through the thoracic duct. The oozing point was seized and ligatured. The glands were then peeled from the third part of the subclavian artery, and when this was accomplished their removal from the posterior triangle and from under the anterior border of the trapezius was easily accomplished. The ends of the sterno mastoid were sutured and the wound drained and dressed. The tube was removed on the third day, and the wound healed absolutely by first intention, no ill effects being noted from the division of the thoracic duct.

CASE 2.—A boy, W. M., age 16, was brought under my notice last June, with a large tumor occupying the left side of the neck, dipping deeply under the mastoid origin of sterno mastoid, and completely filling the posterior triangle. The surface of the tumor was nodular, but no soft spots were observed, the whole mass having a uniform consistence. Glandular enlargement co-existed in both axillæ. No enlargement of liver and spleen could be made out. An examination of the blood showed a decided increase of white blood corpuscles, and this added to the fact that the temperature was normally elevated every evening, led me to think the case was one of lymphadenoma. The glands, however, were not isolated sufficiently, nor were they freely movable enough on the deeper structures to be absolutely certain; added to which there was distinct adhesion between the growth and the deep vessels. The case was treated by injections of five minims of Fowler's solution of arsenic every other day, with nourishing diet and cod liver oil. The second injection was followed by an acute attack of inflammation, which subsided under the use of hot fomentations and rest; but the glands still continuing to enlarge, operation was decided on.

The usual incision was made along the anterior border of the sterno mastoid, extending from mastoid process to sternum, with a cross incision at right angles, across the posterior triangle to the anterior border of the trapezius muscle. The sterno mastoid was cut through and the growth exposed, with the posterior belly of omo-hyoid tightly stretched over it. This was divided and search made mesially for the carotid artery and internal jugular vein. While working at this margin of the tumor, the scalpel suddenly passed into the jugular vein which formed part of the capsule. We turned our attention to this vessel at the upper thoracic aperture, and there isolated and ligatured it. The tumor was now carefully removed from the supra-clavicular space to the subclavian artery and vein exposed. The phrenic nerve, covered by a thin layer of fascia, came into view, while great care had to be used in peeling the glands from the cords of the brachial plexus. At the upper end of the wound the tumor and vein, still adherent, were removed from the common carotid artery and vagus nerve which were cleanly dissected, while a prolongation of the tumor, running along the facial and superior thyroid veins, gave a great deal of trouble. The most difficult part of the operation, however, was the removal of the upper part of the tumor which dipped under the posterior belly of the digastric and the stylo hyoid, still firmly adherent to internal jugular. By forcibly pulling these muscles upwards, we managed to pass a ligature around the vein above the growth, which was then removed in toto. All tubercular material was then removed from under surface of the sterno mastoid, the divided ends sutured, and the wound drained behind and closed. At the anterior part of the incision the wounded superior thyroid vein eluded all efforts to pick it up, and the cavity was packed with iodoform gauze to prevent oozing.

The case progressed favorably; there was a little shock, but the patient left the hospital within a week. The wound suppurated, and from time to time small collections of pus had to be evacuated. At the present time the wound in the neck is closed, but there is a slight enlargement of the lymphatic glands at the angle of the jaw and under the chin. During the removal of the upper part of the growth, the parotid gland was cut into, and a twig from the facial supplying the "depressor anguli oris," divided. Examination of the glands showed marked caseation.

Case 3.—W. M., colored, aged 24, applied for relief for a tumor occupying the upper part of the right side of the neck, dipping deeply under the origin of the sterno mastoid and extending to within two inches of the clavicle. The tumor was globular and non-fluctuating, and was firmly fixed to the underlying tissues. There was no anæmia, no enlargement of liver or spleen, and no alteration in the character of the blood. A diagnosis of tubercular glands was made, and an operation demanded.

The usual incision along the anterior border of the sterno mastoid was employed. While elevating that structure from the growth, a large abscess cavity was opened. The common carotid and jugular vein were exposed, and the growth separated from them fairly easily except at the upper part of the incision. With care, however, the growth was separated from its important anterior relations, but not before the spinal accessory nerve had been divided and the vagus nerve and superior cervical ganglion of the sympathetic freely exposed to view. The upper portion and the greater part of the lower portion of the sterno mastoid being implicated in the disease were removed, the wound drained and closed. The case progressed very satisfactorily, healing being completed within a week.

I have not been able to follow the case since, but I judge that all is well.

CASE 4.—I. B., colored, aged 25, two years ago applied for relief on account of a large tumor occupying the left side of the neck, occupying the upper part of the posterior triangle of the neck. It was globular and firmly fixed to the deeper structures. There was no anæmia and no enlargement of liver or spleen, nor was the blood altered.

I made the usual incision along the anterior border of the sterno mastoid, and finding the growth adherent to the vessels above, threw a silk ligature around the common carotid to control hemorrhage. This was not tied, but my assistant compressed the vessel against the loop with his finger. The glands which were found to be tubercular, were removed without much difficulty, as the adhesions to the sheath of internal jugular vein were loose. The wound was drained, closed, and healed up in a few days.

CASE 5.—A man, aged 35, white, was admitted under my care having pedunculated malignant growth of the left vocal cord, which had been causing attacks of intense dyspnœa for about six months, suffocation being imminent during the attacks. He had a mass of gland on the left side of the neck about the size of a tangarine orange. This was firmly adherent to deep structures, presumably to the deep vessels. After consultation I determined to remove the cervical growth and simultaneously the left half of the larynx, although this was a serious undertaking in the patient's weak state.

An incision was made along the anterior border of the sterno mastoid and the growth exposed. While attempting its removal the jugular vein was opened and we had great difficulty in securing the bleeding point. After a tedious operation, lasting fully an hour, the growth, together with three inches of vein were removed, but the patient was too exhausted to allow us to proceed further. I closed the wound and dressed it, intending to excise the larynx at a subsequent operation, but our patient, about five days afterwards, nearly choked in the ward, necessitating an immediate tracheotomy. Broncho pneumonia set in and he died in about ten days.

The diagnosis of glandular tumors in the neck is in the great majority of cases an easy matter, but occasionally one meets with cases presenting insuperable difficulties. Usually confusion arises between lymphadenoma and tubercle, for in both we may have, as I have shown, an increase of the white blood cells, and a rise of temperature in the evening and a certain degree of anæmia. In the latter stages of lymphadenoma the liver and spleen become involved, but the absence of this symptom never discredits a diagnosis. Lympho-sarcoma usually grows rapidly, implicates the deeper structures of the neck when it is absolutely fixed, and sooner or later implicates the skin. I have more than once refused to operate on these cases because I felt morally certain that the growth had surrounded both the carotid artery and the vagus nerve, and any procedure would either be incomplete and partial or would end in disaster. The earlier stages of lympho-sarcoma might be subjected to radical treatment, but as far as my experience goes, I have never seen one in such a stage.

Having made up one's mind to operate, there are many points which require careful consideration:

### I. THE SKIN INCISION.

Undoubtedly the best place is the anterior border of the sterno mastoid, closely hugging that muscle, if it is necessary to prolong the incision as high as the tip of the mastoid process. By deviating from this at the upper end it is easy to wound the facial twigs supplying the lower lip, which has happened to me twice.

### II. THE DEEPER PART OF THE INCISION.

The second part of the operation should be directed to isolating the carotid artery and jugular vein. *This is the key to the whole operation*, and if the growth can be separated along its whole length, it can be forcibly pulled backward and removed from the posterior triangle, with little risk of injuring any portion of the structures. The glands can be dissected away back best with closed forceps; in fact, we rather during the early stages dissect the important vessels and nerves from the glands, leaving them undisturbed for a time. The vagus nerve must be preserved, but with care it is not liable to injury. No hesitation should be held about dividing the sterno mastoid, and even, if affected, about removing it "in toto," as the movements of the neck will in no way be interfered with by its absence.

In regard to hemorrhage, the most troublesome is that from the internal jugular vein. Unless one works with a free incision and with plenty of room, a wound of the jugular would prove very troublesome; and very often, by isolating the vein above and below and ligaturing it in both places, the tumor can be removed bloodlessly, and with great rapidity.

Arterial hemorrhage need give rise to no anxiety. At the upper part of the neck the occipital and posterior auricular arteries are the ones likely to be injured, as they course backward; the anterior branches of the external carotid are out of the way altogether, except in instances of implication of the submaxillary and submental lymph glands. It is quite unnecessary to put even temporary ligature around the carotid, except in extensive implication of the latter. In fact, the larger our experience, the less we need such help.

As regards muscles, as I have said before, the sterno mastoid can be removed with impunity; there is also nothing gained by attempting to save the omo hyoid. In cases where the glands extend high up underneath the posterior belly of the digastric it might be advisable to divide the muscle, but I have usually, found that it can be retracted without difficulty.

Nerves should be preserved as far as possible, particularly the vagus, phrenic and sympathetic nerves. The spinal accessory may be removed with impunity. I have never, so far, had occasion to see the hypoglossal or the superior laryngeal, but it would be as well to remember their anatomical position when following up glands among the branches of the external carotid. Injury to the thoracic duct seems never to be followed by any deterioration of strength. I watched case 1 very carefully to see whether there would be a chylus fistula or any cachexia, but neither resulted. For further information on wounds of the thoracic duct, an interesting paper, by W. W. Keen,* might be consulted.

---

*New York Medical Journal, May, 1894.

There is no class of operation where a knowledge of anatomy is more essential, and this must not be hearsay knowledge, but the surgeon must have an accurate mental picture of the exact relations of every structure long before it is exposed; and it is here also that a good anatomist might quickly, unless he were accustomed to meet surgical contingencies, give credence to the often quoted saying that "a good anatomist makes a bad surgeon.'

